# Key Risk Factors Associated With Electronic Nicotine Delivery Systems Use Among Adolescents

**DOI:** 10.1001/jamanetworkopen.2023.37101

**Published:** 2023-10-20

**Authors:** Thuy T. T. Le

**Affiliations:** 1 Department of Health Management and Policy, School of Public Health, University of Michigan, Ann Arbor

## Abstract

**Importance:**

The prevalence of electronic nicotine delivery systems (ENDS) use among US youths has increased significantly during the past decade. Identifying key factors highly associated with ENDS use is essential in monitoring and preventing this harmful behavior among youths.

**Objective:**

To identify the most important risk factors in wave 4.5 (ie, December 2017 to December 2018) of the Population Assessment of Tobacco and Health Study (PATH) data that are associated with ENDS use in wave 5 (ie, December 2018 to November 2019) among adolescents who were tobacco-naive at baseline.

**Design, Setting, and Participants:**

This prognostic study examined data from waves 4.5 and 5 of the PATH youth data set using machine learning techniques. The PATH study is a nationally representative longitudinal cohort study of tobacco use and health in the United States among individuals aged 12 years and older. The data analysis was carried out between January and April 2023.

**Main Outcomes and Measures:**

Wave 5 current ENDS use status of wave 4.5 adolescents who were tobacco-naive.

**Results:**

The analyzed data set comprised 7943 individuals who were tobacco-naive in wave 4.5. Among this group, 332 participants (4.2%) indicated their present use of ENDS in wave 5, 5047 (63.5%) were aged 12 to 14 years, 4066 (51.2%) were male, and 2455 (30.9%) were Hispanic. The most important risk factors of ENDS use in wave 5 among adolescents who were tobacco-naive in wave 4.5 were the likelihood of using ENDS if offered by a best friend (mean SHAP value, 0.184), the number of best friends using e-cigarettes (mean SHAP value, 0.167), household tobacco usage (mean SHAP value, 0.161), curiosity about ENDS use (mean SHAP value, 0.088), future intention to use ENDS (mean SHAP value, 0.068), youth’s total average weekly earnings (mean SHAP value, 0.060), and perceptions of tobacco product safety (mean SHAP value, 0.026).

**Conclusions and Relevance:**

The findings of this study suggest that family and friends play an important role in ENDS use among adolescents. The top-ranking factors associated with ENDS use in this study are areas for further exploration, given the increasing prevalence of ENDS use among youths in recent years. Additionally, these findings highlight the important role of families and schools in shaping adolescents’ tobacco-related knowledge, which can protect them from using ENDS.

## Key Points

**Question:**

What are the key variables in wave 4.5 (ie, December 2017 to December 2018) of the Population Assessment of Tobacco and Health Study associated with the use of electronic nicotine delivery systems (ENDS) in wave 5 (ie, December 2018 to November 2019) among adolescents who had never used tobacco at baseline?

**Findings:**

In this prognostic study of 7943 adolescents, the most important factors associated with ENDS use in wave 5 among adolescents who were tobacco-naive in wave 4.5 were the likelihood of using ENDS if offered by a best friend, having best friends who use ENDS, living with a person who uses tobacco, being curious about ENDS, expressing an intention to use ENDS, average weekly income, and perception of the safety of tobacco products.

**Meaning:**

These findings suggest that family and friends are important factors when analyzing adolescent risk for ENDS use.

## Teaser

This prognostic study examines factors associated with electronic nicotine delivery systems use among adolescents who had never used tobacco using data from the Population Assessment of Tobacco and Health Study.

## Introduction

The prevalence of electronic nicotine delivery systems (ENDS), including e-cigarettes, e-hookahs, e-pipes, and vape pens, among US youths (12 to 17 years), has increased significantly during the past decade.^1^ ENDS have been the youth’s most used tobacco product. In 2021, approximately 2.8% of middle school students and 11.3% of high school students used electronic cigarettes in the past 30 days, according to the National Youth Tobacco Survey.^2^ In 2018, the Surgeon General declared a youth e-cigarette epidemic.^1^ Individuals who use ENDS are exposed to harmful substances, which potentially pose health risks, in addition to nicotine.^3^ Nicotine addiction may negatively impact youth’s brain development and psychosocial health. Furthermore, for some individuals, ENDS use at a young age may act as a gateway to initiating other tobacco products, including conventional cigarettes.^3^ As such, identifying key factors highly associated with ENDS use is essential in monitoring and preventing this harmful behavior among youths.

Machine learning (ML) algorithms are a powerful tool to handle high-dimensional data with multicollinearity and complex interactions to predict outcomes with high accuracy and effectively identify hidden patterns in the data.^4^ As such, ML methods can address some limitations of traditional statistical methods, as discussed in Le et al.^5^ These abilities of ML methods make them promising candidates when dealing with complex survey data, such as the Population Assessment of Tobacco and Health (PATH) survey. A growing body of research studies applies ML methods to local and national survey data to investigate tobacco use behaviors. Fu et al^6^ used local survey data in California to build a random forest to identify the top risk factors of vaping initiation. Han et al^7^ trained a penalized linear regression to discover emerging risk factors of ENDS use in the PATH waves 1 to 4. Le et al^5^ used random forest–recursive feature elimination (RF-RFE) to systematically uncover unanticipated risk factors of smoking initiation in the PATH data by analyzing the PATH waves 1 to 2 and waves 4 to 5. Previous studies^6,7^ have used only a set of manually selected survey variables to train ML models and analyze the most important variables among those selected. Le et al^5^ has been one of the first few studies in tobacco control that used ML techniques to automatically acquire a set of the most informative variables of smoking initiation among all possible variables in the national representative PATH survey. In doing so, the authors discovered unanticipated risk factors of these behaviors, such as body mass index (BMI, calculated as weight in kilograms divided by height in meters squared) and dental or oral health status.

The present study used a combination of ML techniques, including RF-RFE, the eXtreme Gradient Boosting method (XGBoost), and Shapley Additive Explanation (SHAP). The objective was to systematically identify the most significant risk factors among all wave 4.5 PATH variables that demonstrate strong associations with ENDS use in wave 5 among adolescents who were tobacco-naive in wave 4.5. Compared with Han et al,^7^ this study used more contemporary data, specifically waves 4.5 to 5, while Han et al^7^ analyzed waves 1 to 4. Due to the rapidly changing landscape of tobacco products, unanticipated risk factors of ENDS use are likely to emerge over time. Analyzing the most updated data would provide tobacco regulators and researchers with the latest information. In addition, this study used a combination of ML methods to automatically extract a set of the most important risk factors of ENDS use from all possible PATH variables for the development of XGBoost, instead of manually selecting a subset of PATH variables to train a penalized linear regression as in Han et al.^7^ In doing so, unanticipated variables associated with ENDS use among adolescents who were tobacco-naive were less likely to be missed. This ML-based approach offers tobacco researchers and regulators a novel tool for gaining valuable insights into tobacco use behaviors, which play a crucial role in combating the tobacco epidemic more effectively.

## Methods

This prognostic study followed the Transparent Reporting of a Multivariable Prediction Model for Individual Prognosis or Diagnosis (TRIPOD) reporting guideline and was approved by the institutional review board at the University of Michigan. Informed consent was waived because the study uses data that are publicly available and deidentifed.

### Data

The PATH study is a nationally representative longitudinal survey designed to monitor tobacco use and its health effects among US youths and adults.^8^ This ongoing study collects individual-specific information, including demographics, physical and mental health, tobacco use behaviors, and tobacco risk perception, among others. It is conducted annually or biannually, with its first wave released in 2013. More details of this survey can be found on the US Food and Drug Administration’s website.^8^ This study focused on analyzing the youth PATH waves 4.5 (ie, December 2017 to December 2018) and wave 5 (ie, December 2018 to November 2019). Here, I was interested in predicting the current ENDS use status (past 30-day ENDS use) in the following wave among individuals aged 12 to 16 years in the US who were tobacco-naive at baseline using variables from the baseline wave. ENDS were defined as e-cigarettes, vape pens, personal vaporizers and mods, e-cigars, e-pipes, e-hookahs, or hookah pens. Tobacco-naive individuals were defined as respondents who reported to have never smoked cigarettes, traditional cigars, cigarillos, filtered cigars, pipe, hookah, bidis, or kretek and have never used e-products, smokeless tobacco, snus pouches, or dissolvable tobacco.

### Statistical Analysis

Wave 4.5 served as the baseline wave to predict the use of ENDS in wave 5. The outcome is the current ENDS use status in wave 5, 1 year after baseline wave 4.5. A respondent’s current ENDS use status is yes if they have used ENDS within the past 30 days, and no if they have not used ENDS within the past 30 days. Among 13 131 adolescents in wave 4.5, 9650 were tobacco-naive, of whom 7943 had nonmissing ENDS use status and had not aged out in wave 5. In this study, individuals were excluded if they were aged 17 years at baseline since they would be aged 18 years and respond to the adult questionnaire instead of the youth questionnaire in the follow-up wave. As a result, the sample size is 7943 for waves 4.5 to 5. To consider only meaningful variables, variables irrelevant to the research question were removed (eg, random questions, imputed variables, youth types, status of collected specimens, etc).

**Figure 1.**
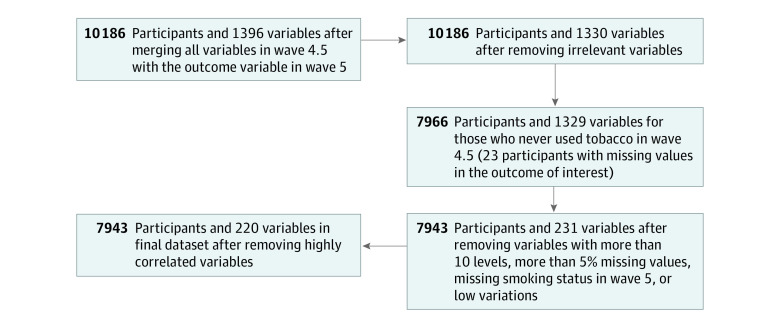
A Diagram of Preliminary Data Processing

Furthermore, highly associated variables (Cramer's V effect size ≥ 0.75) and those with more than 5% missing values were removed. The remaining missing values were imputed using the rfImpute function of the randomForest package in R (R Project for Statistical Computing)^9^ to maximize the analysis sample size. Figure 1 provides a detailed description of this data processing. The final clean data set consisted of 7943 individuals who were tobacco-naive in wave 4.5. Among these individuals, 332 participants (4.2%) reported current ENDS use in wave 5. This data set comprised 219 wave 4.5 variables used in the subsequent analysis (eTables 1 and 2 in Supplement 1).

The clean data set was divided into training data (80%) and testing data (20%) so that the distribution of the outcome variable in each data set remains as in the whole set. The training data were used for feature selection, hyperparameter optimization, and model calibration, whereas the testing data were used exclusively for evaluating model performance on unseen data. Different ML algorithms addressed the study’s research question. Initially, the most informative risk factors for ENDS use were extracted from all available PATH variables in baseline wave 4.5. Subsequently, the ML model was trained on the selected wave 4.5 variables to evaluate how well the ENDS use status in wave 5 can be predicted. Finally, these selected variables were ranked according to their contributions to the model’s prediction.

#### Feature Selection

After preliminary data preprocessing, a set of 219 wave 4.5 variables was obtained. To enhance model performance and identify key risk factors for future ENDS use, I applied RF-RFE, a widely used feature selection technique using recursive feature selection based on random forest,^10,11^ to select the most informative risk factors associated with ENDS use in wave 5 from the pool of wave 4.5 variables.

To obtain the final set of variables, I repeated the RF-RFE process until the intersection of the optimal feature subsets produced by all iterations remained stable. In each iteration of RF-RFE, RF-RFE with cross-validation (4 folds, repeated 5 times) was trained on the training data to identify the optimal feature subset. To address the challenge posed by class imbalance, random oversampling examples through the ROSE package version 0.0-4 in R (R Project for Statistical Computing)^12^ were used within each cross-validation iteration. Specifically, oversampling was applied exclusively to the training folds while keeping the validation fold unchanged. In each iteration, the optimal feature subset was determined once the classification accuracy reached 99% of the highest accuracy. The ultimate and most important subset of variables comprised the RF-RFE selected features, consistently chosen in at least 95% of all simulations. All of these steps were carried out using the training data. Le et al^5^ has more details on this feature selection process.

#### Model Development

Gradient boosting is an ensemble ML algorithm that is developed by subsequently adding weak models (ie, decision trees) to the ensemble to minimize the error produced by the previous models.^13,14^ XGBoost, a scalable and highly accurate implementation of gradient boosting,^14^ has been widely used for accomplishing cutting-edge performance in numerous ML tasks. Several hyperparameters of XGBoost can be tuned to improve the model performance. Here, bayesian optimization^15^ was used to search for the optimal values of the following hyperparameters: learning rate control (LRC), maximum depth of a tree (MDT), minimum sum of instance weight (hessian) needed in a child (MCW), subsample ratio of the training instance (SS), and the subsample ratio of columns when constructing each tree (CT), in a given parameter space. A full description of these hyperparameters can be found in the XGBoost documentation.^16^ To handle this class-imbalanced data set, the hyperparameter weight balance control (WBC) was also optimized as part of the parameter tuning process. The wave 4.5 to wave 5 training data with the RF-RFE selected features were utilized to train XGBoost and optimize its hyperparameters to predict the current ENDS use status in wave 5.

#### Variable Importance

Comprehending the reasoning behind the predictions made by most ML models is often challenging, if not impossible. Thus, Shapley additive explanations (SHAP), a technique stemming from cooperative game theory, was introduced by Lundberg and Lee in 2017.^17^ This approach has been widely adopted in the literature to provide insights into complex ML models.^5,18,19^ SHAP values are computed to explain the contribution of each risk factor to the final model prediction. Here, the RF-RFE selected risk factors are ranked based on their SHAP values, representing their impact on the model’s output. To ensure robust SHAP values for all risk factors, the XGBoost model was trained 1000 times using the training data set. The final SHAP value for each risk factor was computed by computing the mean of the 1000 SHAP values. I opted for 1000 iterations because additional iterations did not significantly alter the relative rankings of these selected risk factors.

## Results

The data set under analysis included 7943 individuals who were tobacco-naive in wave 4.5. Within this group, 332 individuals (4.2%) reported using ENDS in wave 5. Among the 7943 individuals who were tobacco-naive in wave 4.5, 5047 (63.5%) were between the ages of 12 and 14 years, 4066 (51.2%) were male, and 2455 (30.9%) identified as Hispanic (eTable 3 in Supplement 1).

**Figure 2.**
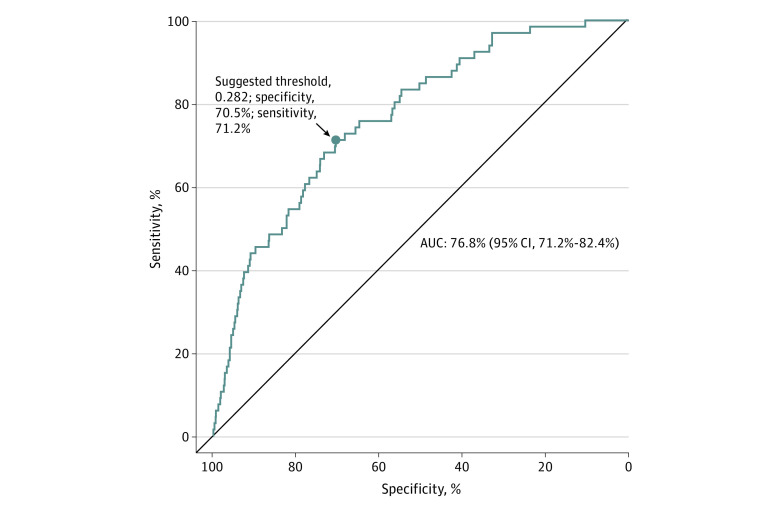
A Receiver Operating Characteristic Curve of the XGBoost Classifier AUC indicates area under the curve and is presented along with a suggested threshold of specificity and sensitivity.

The feature selection process resulted in 44 most informative variables from wave 4.5. Using the training data from waves 4.5 to 5 with the RF-RFE selected variables, the optimal values of 5 XGBoost hyperparameters (LRC, MDT, MCW, SS, CT, and WBC) are shown in Table 1. With these selected variables and the wave 4.5 to 5 testing data set, the XGBoost model produced highly accurate predictions of the current ENDS use status in wave 5 (area under the curve [AUC], 77%; 95% CI, 71%-82%) (Figure 2).

**Table 1.** The Parameter Space and Optimal Values of the Optimized XGBoost Hyperparameters

Hyperparameters	LRC	MDT	MCW	SS	CT	WBC
Parameter space	0-0.25	1-6	1-6	0.5-1	0.5-1	10-25
Optimal value	0.06	2	1.17	0.94	0.63	12.14

Abbreviations: CT, subsample ratio of columns when constructing each tree; LRC, learning rate control; MCW, minimum sum of instance weight (hessian) needed in a child; MDT, maximum depth of a tree; WBC, weight balance control; SS, subsample ratio of the training instance.

**Figure 3.**
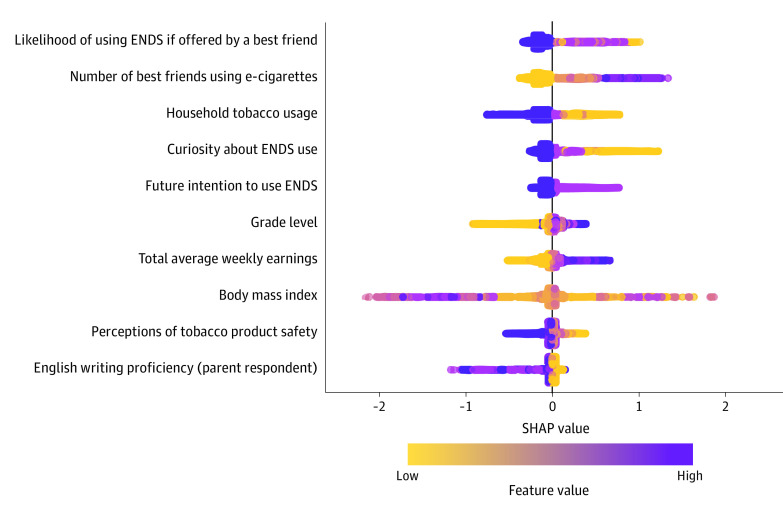
The Top 10 Most Informative Variables in Waves 4.5 and 5 ENDS indicates electronic nicotine delivery systems. Body mass index is calculated as weight in kilograms divided by height in meters squared. The mean SHAP values for each variable can be found in Table 2.

Table 2 and Figure 3 present the descriptions and rankings of the top 10 selected variables according to their mean SHAP values (ie, their contribution to the model’s prediction). The significance of the association between a risk factor and the ENDS use status in wave 5 increases as its mean SHAP value rises. These top 10 most important variables include the likelihood of using ENDS if offered by a best friend (mean SHAP value, 0.184), the number of best friends using e-cigarettes (mean SHAP value, 0.167), household tobacco usage (mean SHAP value, 0.161), curiosity about ENDS use (mean SHAP value, 0.088), future intention to use ENDS (mean SHAP value, 0.068), grade level (mean SHAP value, 0.068), youth’s total average weekly earnings (mean SHAP value, 0.060), BMI (mean SHAP value, 0.055), perceptions of tobacco product safety (mean SHAP value, 0.026), and English writing proficiency (parent respondent) (mean SHAP value, 0.024).

**Table 2.** Description of the Top 10 Most Important Variables of Current ENDS Use Status in Wave 5 With the Mean SHAP Values

Order	Variable	Variable description (value) in PATH	Mean SHAP value
1	Likelihood of using ENDS if offered by a best friend	Would use an ENDS product if one of your best friends offered you one (1, definitely yes; 2, probably yes; 3, probably not; 4, definitely not)	0.1836
2	No. of best friends using e-cigarettes	How many of your best friends use e-cigarettes (1, none; 2, a few; 3, some; 4, most; 5, all)	0.1667
3	Household tobacco usage	Anyone who lives with you now uses tobacco (1, cigarettes, cigars, cigarillos or filtered cigars; 2, e-products exclusively; 3, other tobacco products, including smokeless, snus and hookah; 4, no one living in the home uses tobacco)	0.1607
4	Curiosity about ENDS use	Ever been curious about using an ENDS product (1, very curious; 2, somewhat curious; 3, a little curious; 4, not at all curious)	0.0879
5	Future intention to use ENDS	Think you will use ENDS in the next year (1, definitely yes; 2, probably yes; 3, probably not; 4, definitely not)	0.0680
6	Grade level	Grade level (if on holiday or break, use grade level entering when returning to school) (1, 7th grade or younger; 2, 8th grade; 3, 9th grade; 4, 10th grade; 5, 11th grade; 6, other, including not enrolled this year or last, home schooled, school not graded, 12th grade, college, or vocational)	0.0679
7	Total average weekly earnings	Money received in total during an average week (1, none; 2, less than $1; 3, $1 to $5; 4, $6 to $10; 5, $11 to $20; 6, $21 to $50; 7, $51 to $100; 8, $101 to $150; 9, $151 or more)	0.0601
8	BMI	Wave 4.5 youth BMI (mean [SD], 22.92 [5.44]; minimum: 9.74; maximum, 79.09)	0.0554
9	Perceptions of tobacco product safety	Agree or disagree: some tobacco products are safer than others (1, strongly agree; 2, agree; 3, neither agree nor disagree; 4, disagree; 5, strongly disagree)	0.0258
10	English writing proficiency (parent respondent)	How well you write in English (parent respondent) (1, very well; 2, well; 3, not well; 4, not at all)	0.0238

Abbreviations: BMI, body mass index (calculated as weight in kilograms divided by height in meters squared); ENDS, electronic nicotine delivery system; PATH, Population Assessment of Tobacco and Health Study; SHAP, Shapley Additive Explanation.

In Figure 3, the beeswarm plot displays the SHAP values for each risk factor for each data point, revealing the directional impact of each feature’s values on the model output. Due to XGBoost requirements, respondents’ current ENDS use status was recoded as 1 if they had used ENDS within the past 30 days and 0 if they had not. The values of the risk factors with positive (negative) SHAP values are linked to an increase (decrease) in the likelihood of ENDS use in wave 5. For example, having more friends using e-cigarettes is associated with positive SHAP values, indicating a higher likelihood of ENDS use. Conversely, declining an offer of ENDS from a best friend reduces the outcome value, indicating a lower likelihood of ENDS use. The interpretation of the remaining top 10 variables is similar, using the detailed information in Table 2 and Figure 3. eTable 4 and the eFigure in Supplement 1 contain a comprehensive list of all 44 selected variables with their mean SHAP values and descriptions.

## Discussion

With the help of ML techniques, 44 variables were extracted from wave 4.5. These variables were associated with the current ENDS use status in wave 5 among adolescents who were tobacco naive in wave 4.5. The findings show that XGBoost is able to classify the status of current ENDS use in the outcome wave with high accuracy for waves 4.5 to 5 (AUC, 77% [95% CI, 71%-82%]). This study strengthens the role of ML algorithms in predicting future tobacco use behaviors and uncovering important factors of these behaviors in large survey data.

The top 3 most important variables associated with ENDS use in the following wave are the likelihood of using ENDS if offered by a best friend, the number of best friends using e-cigarettes, and household tobacco usage (Table 2 and Figure 3). Adolescents who were tobacco-naive and had best friends who use ENDS, who responded that they would try ENDS if their best friends offered one, or who lived with a person who uses tobacco are more likely to start using ENDS in the near future. These results align with previous studies^20,21,22^ that have highlighted the association of family and friends with ENDS use. However, the prominence of these factors as the top 3 variables raises concerns regarding their significant association with ENDS use. During waves 4.5 to 5 (ie, from 2017 to 2019), the tobacco landscape underwent a drastic transformation with the rapid rise of vaping among adolescents.^23^ ENDS use among youths was declared as an epidemic by the US Surgeon General in 2018.^1^ The identification of these top-ranked risk factors may suggest their potential role in accelerating the widespread use of ENDS during this critical period.

Among the top 10 variables, adolescents’ total average weekly earnings show a significant association with future ENDS use. Its SHAP values indicate that a higher average weekly income is associated with a higher risk of current ENDS use after a 12-month follow-up among youths. A positive association between adolescents’ personal income and cigarette smoking has also been reported in the literature.^24,25^ Adolescents with high weekly income likely earn it from their employment, which suggests it could be an indirect measure of the amount of time they spend at work. Previous studies have found a positive correlation between the amount of time spent working for pay and tobacco use.^26^ While there could be other explanations for this association, in light of these findings, closely monitoring adolescents’ spending habits and daily activities may help prevent them from engaging in undesirable behaviors.

Other noteworthy variables among the top 10 are curiosity about ENDS use and future intention to use ENDS. Adolescents who were curious about ENDS use or express an intention to try it soon were more susceptible to using ENDS. These results are consistent with those in Kong et al,^22^ which identified curiosity as a leading reason for ENDS experimentation among adolescents and young adults. Hence, pinpointing the determinants associated with these psychological factors can provide valuable insights for designing future tobacco control strategies. Furthermore, adolescents’ perceptions of tobacco product safety were associated with their future ENDS use. Perceiving all tobacco products as unsafe was associated with a lower risk of using ENDS. These findings underscore the critical role of families and schools in shaping youth’s tobacco-related knowledge to protect them from these harmful products.

Similar to Le et al,^5^ the present study suggests that several variables, including BMI, oral and dental health, exposure to radio and television, mental and physical health status, and tobacco risk perceptions and awareness, are associated with current ENDS use in wave 5 among adolescents who were tobacco-naive in wave 4.5 (eTable 4 and the eFigure in Supplement 1). This provides further evidence regarding the association of these factors with tobacco use behaviors.

This analysis used a set of PATH variables automatically selected by RF-RFE from all possible PATH variables in wave 4.5. In contrast, Han et al^7^ manually selected a set of variables from waves 1 to 3 based on their expert knowledge and the existing literature. Thus, different sets of variables were identified in general. However, the number of best friends using e-cigarettes and household tobacco usage are among the top 10 variables associated with ENDS use in the present study and in wave 4 in Han et al.^7^ This consistency highlights the robust influence of peers and family on youth’s ENDS use. However, the remaining variables in this study’s top 10 were not included in Han et al.^7^ Among the top 10 variables linked to ENDS use in wave 4 in Han et al,^7^ hours spent watching television in a typical day and parental education attainment were found to be important in the present study but are not among the top 10 variables. The similarities in findings between these studies suggest the consistent association of certain factors with ENDS use. However, the differences in selected variables suggest that using RF-RFE to search for an optimal set of PATH variables automatically may uncover factors that are overlooked during manual selection, and new factors associated with ENDS use may emerge over time due to the rapid changes in the tobacco product market.

### Limitations

This study has limitations. This work is based on the youth PATH survey data. Therefore, the most informative variables were extracted from all available variables in the PATH data. Thus, other important factors that are not included in these data sets may have been missed. Although the relative rankings of the selected variables were obtained as a mean of 1000 simulations, a sensitivity analysis showed that their rankings may slightly change due to the random nature of the ML algorithms. Different feature selection methods may also suggest different sets of variables. However, these findings are likely to be robust because of the high rankings of these variables. Finally, it is important to note that these findings suggest the associations between risk factors and the outcome variable and do not establish causal relationships, which is inherent to the nature of the ML model used in this study.

## Conclusions

These findings showed significant associations of family and peer influence with current ENDS use after 1 year of follow-up among adolescents who were tobacco-naive at baseline. Given the popularity of ENDS use in the past years, the prominence of these risk factors raises concerns about their potential contribution to the widespread use of ENDS. Furthermore, these findings also underscored the pivotal role of education in shaping adolescents’ awareness of tobacco-related matters, serving as protective measures against these detrimental products.

## Notes

Supplement 1. **eTable 1.** List of 230 Wave 4.5 Variables **eTable 2.** List of Wave 4.5 Variables Removed From eTable 1 Due to High Correlation **eFigure.** Rankings of 44 RF-RFE Selected Variables With Their Mean SHAP Values in Waves 4.5 to 5 **eTable 3.** Baseline Characteristics of the Study Sample **eTable 4.** An Exhaustive List of the Wave 4.5 Variables Together With Their Detailed Description and SHAP Values

Supplement 2.
Data Sharing Statement
