# Supplementary material for: Key Risk Factors Associated With Electronic Nicotine Delivery Systems Use Among Adolescents
Source: JAMA Netw Open. 2023 Oct 20;6(10):e2337101. doi: 10.1001/jamanetworkopen.2023.37101 (PMC10589803; doi:10.1001/jamanetworkopen.2023.37101)
Supplement: Supplement 1. — eTable 1. List of 230 Wave 4.5 Variables eTable 2. List of Wave 4.5 Variables Removed From eTable 1 Due to High Correlation eFigure. Rankings of 44 RF-RFE Selected Variables With Their Mean SHAP Values in Waves 4.5 to 5 eTable 3. Baseline Characteristics of the Study Sample eTable 4. An Exhaustive List of the Wave 4.5 Variables Together With Their Detailed Description and SHAP Values [file jamanetwopen-e2337101-s001.pdf]

Supplemental Online Content

Le TTT. Key risk factors associated with electronic nicotine delivery systems use among adolescents. *JAMA Netw Open*. 2023;6(10):e2337101.
doi:10.1001/jamanetworkopen.2023.37101

eTable 1. List of 230 Wave 4.5 Variables
eTable 2. List of Wave 4.5 Variables Removed From eTable 1 Due to High Correlation
eFigure. Rankings of 44 RF-RFE Selected Variables With Their Mean SHAP Values in Waves 4.5 to 5
eTable 3. Baseline Characteristics of the Study Sample
eTable 4. An Exhaustive List of the Wave 4.5 Variables Together With Their Detailed Description and SHAP Values

This supplemental material has been provided by the authors to give readers additional information about their work.

© 2023 Le TTT. *JAMA Network Open.*

eTable 1. List of 230 wave 4.5 variables

X04R_Y_PT0047	X04_YC1103	X04_YG9026	X04_YX0158_01	X04_YH1143
X04_PT0003	X04_YC1206	X04_YG9047	X04_YX0158_02	X04_YU1143
X04_PT0009	X04_YC1104	X04_YG9048	X04_YX0158_03	X04_YS1143
X04_PT0011	X04_YC1105	X04_YG9098	X04_YX0158_04	X04_YX0741_01
X04_PT0019	X04_YV1103	X04_YG9125	X04_YN0330_12MH	X04_YX0741_02
X04_PT0021	X04_YV1206	X04_YG9027	X04_YN0331_12MH	X04_YX0741_03
X04_PR1045	X04_YV1104	X04_YG9054	X04_YX0708_01	X04_YX0741_04
X04_PR1051	X04_YV1105	X04_YG9055	X04_YX0708_02	X04_YX0741_05
X04_PR1050	X04_YV9041	X04_YG9099	X04_YX0708_03	X04_YX0741_06
X04_PT0029	X04_YV9042	X04_YG9126	X04_YX0708_04	X04_YX0741_07
X04_PT0035	X04_YZ1002	X04_YH1125	X04_YX0708_05	X04_YX0741_08
X04_PT0033	X04_YR0142	X04_YH9044	X04_YX0708_06	X04_YX0741_09
X04_PT0052_12M	X04_YR0005	X04_YH9110	X04_YX0708_07	X04_YX0741_10
X04_PT0049	X04_YR0110	X04_YH1180	X04_YX0708_08	X04_YX0680
X04_PX0221	X04_YR0115	X04_YH1099	X04_YX0678	X04_YX0681
X04_PX0222	X04_YR0120	X04_YH1124	X04_YX0182	X04_YX0683
X04_PX0707	X04_YR0125	X04_YU1125	X04_YX0203_01	X04_YX0693
X04_PX0731	X04_YR0130	X04_YU1170	X04_YX0203_02	X04_YX0685
X04_PX0732	X04_YR0135	X04_YU1180	X04_YX0203_03	X04_YX0714
X04R_Y_PM0069	X04_YR0206	X04_YU1099	X04_YX0203_04	X04_YX0694
X04R_Y_PM0065_V2	X04_YR0060	X04_YU1124	X04_YX0203_05	X04_YX0500
X04_PM0066	X04_YN0250	X04_YS1125	X04_YX0203_06	X04_YX0200
X04_PM0070	X04_YX0667	X04_YS1170	X04_YX0203_07	X04_YX0715
X04_PM0073	X04_YX0723	X04_YS1180	X04_YX0203_08	X04_YX0716
X04_PM0071	X04_YX0724	X04_YS1099	X04_YX0203_09	X04_YX0499
X04R_Y_PM0130	X04_YC9050	X04_YS1124	X04_YX0203_10	X04_YX0501
X04R_Y_PL0040	X04_YC1149	X04_YX0759	X04_YX0709	X04_YX0502
X04_PX0691	X04_YC1125	X04_YX0494	X04_YX0481	X04_YX0161
X04_PN0335_01	X04_YC1170	X04_YX0474	X04_YX0677_01	X04_YX0162
X04_PN0335_02	X04_YC1180	X04_YX0060	X04_YX0677_02	X04_YX0163
X04_PN0335_03	X04_YC9047	X04_YX0061	X04_YX0677_03	X04_YX0164
X04_PN0335_04	X04_YC9040	X04_YX0062	X04_YX0677_04	X04_YX0165
X04_PN0335_05	X04_YC9118	X04R_Y_YX0671	X04_YX0677_05	X04_YX0166
X04_PN0335_06	X04_YC1124	X04_YX0068	X04_YX0677_06	X04_YX0167
X04_PN0336_01	X04_YC9043	X04_YX0090	X04_YX0677_07	X04_YX0168
X04_PN0336_02	X04_YC9120	X04_YX0091	X04_YX0677_08	X04_YX0169
X04_PN0337_01	X04_YV1125	X04_YX0088	X04_YX0677_09	X04_YX0250
X04_PN0337_02	X04_YV1149	X04_YX0090_12M	X04_YX0677_10	X04_YX0251
X04_PT0060	X04_YV1180	X04_YX0091_12M	X04_YX0710	X04R_Y_YM0018_V2
X04_PT0061	X04_YV1099	X04_YX0088_12M	X04_YX0777	X04_YM0032
X04_PT0062	X04_YV1148	X04_YX0726	X04_YX0008	X04_YM0008
X04R_P_EVR_ASTHMA	X04_YV1152	X04_YX0241	X04_YX0012	X04R_Y_AGECAT2
X04R_P_OTHPAR_INHH	X04_YV1124	X04_YX0242	X04_YX0071	X04R_Y_AGE_CHECK
X04R_P_PARSP_EDUC	X04_YG9025	X04_YX0243	X04_YC1143	X04R_Y_SEX
X04R_Y_YM0065_V2	X04_YG1180	X04_YX0052	X04_YV1143	X04R_Y_HISP
X04_YM0071	X04_YG9097	X04_YX0053	X04_YG1143	X04R_Y_BMI

© 2023 Le TTT. *JAMA Network Open.*

eTable 2: List of wave 4.5 variables removed from Table A2 due to high correlation

X04_PT0011	X04_YX0677_02
X04_PM0070	X04_YH1143
X04_PM0071	X04_YS1143
X04_YR0120	X04_YX0741_10
X04_YX0708_08	X04R_Y_AGECAT2
X04_YX0203_01	

eFigure 1: Rankings of 44 RF-RFE selected variables with their mean SHAP values in waves 4.5-5.

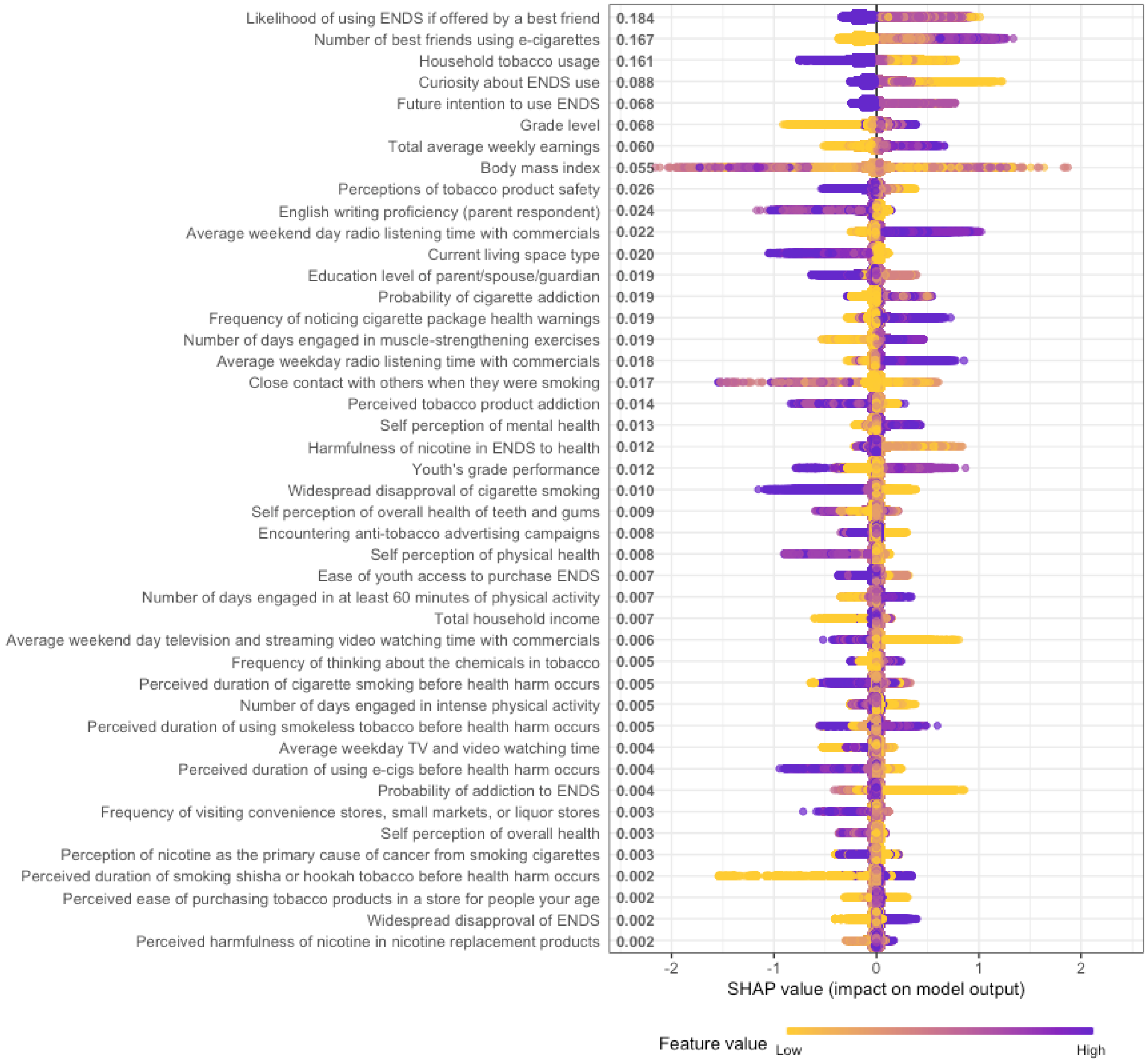

© 2023 Le TTT. *JAMA Network Open.*

eTable 3: Baseline characteristics of the study sample.

Characteristics	Wave 4.5 never tobacco users (N = 7943)	
	Unweighted N	Unweighted %
**Age**		
12 to 14 years old	5047	63.5
15 to 17 years old	2896	36.5
**Sex**		
Female	3877	48.8
Male	4066	51.2
**Hispanic origin**		
Hispanic	2455	30.9
Non-Hispanic	5488	69.1
**Household tobacco use**		
No	5908	74.4
Yes	2035	25.6
**Youth's weekly income**		
$0-$50	7281	91.7
$51-$100	348	4.4
> $100	314	4.0
**School grades**		
Less than mostly B's	2051	25.8
Mostly B's and higher	5892	74.2

© 2023 Le TTT. *JAMA Network Open.*

eTable 4: An exhaustive list of the wave 4.5 variables together with their detailed description and SHAP values.

Variable	Description (Value)	Mean SHAP Value	Scaled SHAP Value (%)
Likelihood of using ENDS if offered by a best friend	X04_YV1105: Would use an electronic nicotine product if one of your best friends offered you one (1 = Definitely yes, 2 = Probably yes, 3 = Probably not, 4 = Definitely not)	0.1836	100.00
Number of best friends using e-cigarettes	X04_YX0681: How many of your best friends use e-cigarettes (1 = None, 2 = A few, 3 = Some, 4 = Most, 5 = All)	0.1667	90.78
Household tobacco usage	X04R_Y_YX0671: DERIVED - Recoded anyone who lives with you now uses tobacco (1 = Cigarettes, cigars, cigarillos or filtered cigars, 2 = E-products exclusively, 3 = Other tobacco products, including smokeless, snus and hookah, 4 = No one living in the home uses tobacco)	0.1607	87.50
Curiosity about ENDS use	X04_YV1103: Ever been curious about using an electronic nicotine product (1 = Very curious, 2 = Somewhat curious, 3 = A little curious, 4 = Not at all curious)	0.0879	47.88
Future intention to use ENDS	X04_YV1206: Think you will use electronic nicotine products in the next year (1 = Definitely yes, 2 = Probably yes, 3 = Probably not, 4 = Definitely not)	0.0680	37.02
Grade level	X04R_Y_YM0018_V2: DERIVED - Recoded grade level (If on holiday or break - grade level entering when returning to school) (6 levels) (1 = 7th grade or below, 2 = 8th grade, 3 = 9th grade, 4 = 10th grade, 5 = 11th grade, 6 = Other (including not enrolled this year or last, home schooled, school not graded, 12th grade, college or vocational)	0.0679	36.96
Total average weekly earnings	X04_YM0008: Money received in total during an average week (1 = None, 2 = Less than $1, 3 = $1 to $5, 4 = $6 to $10, 5 = $11 to $20, 6 = $21 to $50, 7 = $51 to $100, 8 = $101 to $150, 9 = $151 or more)	0.0601	32.73
Body mass index	X04R_Y_BMI: DERIVED - Wave 4.5 Youth Body mass index (Mean: 22.92, Min: 9.74, Max: 79.09, Standard Deviation: 5.44)	0.0554	30.14
Perceptions of tobacco product safety	X04_YX0667: Agree/disagree: Some tobacco products are safer than others (1 = Strongly agree, 2 = Agree, 3 = Neither agree nor disagree, 4 = Disagree, 5 = Strongly disagree)	0.0258	14.03
English writing proficiency (parent respondent)	X04_PM0073: How well you write in English (parent respondent) (1 = Very well, 2 = Well, 3 = Not well, 4 = Not at all)	0.0238	12.95

© 2023 Le TTT. *JAMA Network Open.*

Average weekend day radio listening time with commercials	X04_YX0061: Amount of time spent listening to the radio or online radio stations that include commercials on an average weekend day (1 = None, 2 = Less than 1 hour, 3 = 1 to 2 hours, 4 = 3 to 4 hours, 5 = 5 to 6 hours, 6 = 7 to 8 hours, 7 = 9 to 10 hours, 8 = 11 hours or more)	0.0219	11.95
Current living space type	X04_PX0691: Type of living space where you currently live (1 = Single Family Home, 2 = Duplex, 3 = Double or Multi-Family Home, 4 = Condominium, 5 = Townhouse, 6 = Apartment Building, 7 = None of the above)	0.0197	10.74
Education level of parent/spouse/guardian	X04R_P_PARSP_EDUC: DERIVED - Recoded highest grade or year of school completed by parent/spouse/guardian (6 levels) (1 = Less than High School, 2 = GED, 3 = High school graduate, 4 = Some college (no degree) or associates degree, 5 = Bachelor's degree, 6 = Advanced degree)	0.0193	10.52
Probability of cigarette addiction	X04_YC9043: Likely to become addicted to cigarettes (1 = Very unlikely, 2 = Somewhat unlikely, 3 = Neither likely nor unlikely, 4 = Somewhat likely, 5 = Very likely)	0.0192	10.46
Frequency of noticing cigarette package health warnings	X04_YR0005: In past 30 days, how often noticed health warnings on cigarette packages (1 = Never, 2 = Rarely, 3 = Sometimes, 4 = Often, 5 = Very often)	0.0191	10.38
Number of days engaged in muscle-strengthening exercises	X04_YX0243: In past 7 days, number of days did exercises to strengthen or tone your muscles (1 = 0 days, 2 = 1 day, 3 = 2 days, 4 = 3 days, 5 = 4 days, 6 = 5 days, 7 = 6 days, 8 = 7 days)	0.0186	10.14
Average weekday radio listening time with commercials	X04_YX0060: Amount of time spent listening to the radio or online radio stations that include commercials on an average weekday (1 = None, 2 = Half hour or less, 3 = About 1 hour, 4 = About 2 hours, 5 = About 3 hours, 6 = About 4 hours, 7 = About 5 hours, 8 = About 6 hours, 9 = 7 hours or more)	0.0181	9.86
Close contact with others when they were smoking	X04_YX0068: In past seven days, number of hours that you were in close contact with others when they were smoking (min: 0.00, max: 168.00, mean: 2.05, standard deviation: 9.33)	0.0171	9.29
Perceived tobacco product addiction	X04_YN0250: Believe that nicotine is the main substance in tobacco that makes people want to use tobacco products (1 = Definitely yes, 2 = Probably yes, 3 = Probably not, 4 = Definitely not)	0.0143	7.79
Self perception of mental health	X04_YX0091: Self perception of mental health, including mood and ability to think (1 = Excellent, 2 = Very good, 3 = Good, 4 = Fair, 5 = Poor)	0.0127	6.91

© 2023 Le TTT. *JAMA Network Open.*

Harmfulness of nicotine in ENDS to health	X04_YV1149: Harmfulness of nicotine in electronic nicotine products to health (1 = Not at all harmful, 2 = Slightly harmful, 3 = Somewhat harmful, 4 = Very harmful, 5 = Extremely harmful)	0.0121	6.58
Youth's grade performance	X04_PT0019: In past 12 months, youth's grade performance in school (1 = Mostly A's, 2 = A's and B's, 3 = Mostly B's, 4 = B's and C's, 5 = Mostly C's, 6 = C's and D's, 7 = Mostly D's, 8 = D's and F's, 9 = Mostly F's, 10 = Your child's school is ungraded)	0.0116	6.32
Widespread disapproval of cigarette smoking	X04_YX0008: General perception: Most people disapprove of smoking cigarettes (1 = Definitely yes, 2 = Probably yes, 3 = Probably not, 4 = Definitely not)	0.0104	5.67
Self perception of overall health of teeth and gums	X04_YX0726: Self perception of overall health of teeth and gums (1 = Excellent, 2 = Very good, 3 = Good, 4 = Fair, 5 = Poor)	0.0090	4.89
Encountering anti-tobacco advertising campaigns	X04_YX0777: In past 12 months, how often saw an anti-tobacco advertising campaign (1 = Never, 2 = Rarely, 3 = Sometimes, 4 = Often)	0.0077	4.22
Self perception of physical health	X04_YX0090: Self perception of physical health (1 = Excellent, 2 = Very good, 3 = Good, 4 = Fair, 5 = Poor)	0.0075	4.10
Ease of youth access to purchase ENDS	X04_YX0715: Level of ease for youth to buy e-cigarettes or other electronic nicotine products (1 = Very easy, 2 = Somewhat easy, 3 = Somewhat difficult, 4 = Very difficult)	0.0075	4.06
Number of days engaged in at least 60 minutes of physical activity	X04_YX0241: In past 7 days, number of days physically active for at least 60 minutes per day (1 = 0 days, 2 = 1 day, 3 = 2 days, 4 = 3 days, 5 = 4 days, 6 = 5 days, 7 = 6 days, 8 = 7 days)	0.0071	3.89
Total household income	X04R_Y_PM0130: DERIVED - Recoded total household income in past 12 months (5 levels) (1 = Less than $10,000, 2 = $10,000 to $24,999, 3 = $25,000 to $49,999, 4 = $50,000 to $99,999, 5 = $100,000 or more)	0.0065	3.54
Average weekend day television and streaming video watching time with commercials	X04_YX0474: Amount of time spent watching television and streaming videos that include commercials on an average weekend day (1 = None, 2 = Less than 1 hour, 3 = 1 to 2 hours, 4 = 3 to 4 hours, 5 = 5 to 6 hours, 6 = 7 to 8 hours, 7 = 9 to 10 hours, 8 = 11 hours or more)	0.0064	3.49
Frequency of thinking about the chemicals in tobacco	X04_YR0142: In past 12 months, how often thought about the chemicals contained in tobacco (1 = Never, 2 = Rarely, 3 = Sometimes, 4 = Often, 5 = Very often)	0.0048	2.60
Perceived duration of cigarette smoking before health harm occurs	X04_YC1180: How long you think someone has to smoke cigarettes before it harms their health (1 = It will never harm their health, 2 = Less than a year, 3 = 1 year, 4 = 5 years, 5 = 10 years, 6 = 20 years or more)	0.0047	2.55

© 2023 Le TTT. *JAMA Network Open.*

Number of days engaged in intense physical activity	X04_YX0242: In past 7 days, number of days exercised or participated in physical activity for at least 20 minutes that made you sweat and breathe hard (1 = 0 days, 2 = 1 day, 3 = 2 days, 4 = 3 days, 5 = 4 days, 6 = 5 days, 7 = 6 days, 8 = 7 days)	0.0046	2.53
Perceived duration of using smokeless tobacco before health harm occurs	X04_YS1180: How long you think someone has to use smokeless tobacco before it harms their health (1 = It will never harm their health, 2 = Less than a year, 3 = 1 year, 4 = 5 years, 5 = 10 years, 6 = 20 years or more)	0.0046	2.49
Average weekday TV and video watching time	X04_YX0494: Amount of time spent watching TV or videos on a television, computer, tablet or smartphone on an average weekday (1 = None, 2 = Half hour or less, 3 = About 1 hour, 4 = About 2 hours, 5 = About 3 hours, 6 = About 4 hours, 7 = About 5 hours, 8 = About 6 hours, 9 = 7 hours or more)	0.0044	2.42
Perceived duration of using e-cigs before health harm occurs	X04_YV1180: How long you think someone has to use e-cigarettes before it harms their health (1 = It will never harm their health, 2 = Less than a year, 3 = 1 year, 4 = 5 years, 5 = 10 years, 6 = 20 years or more)	0.0044	2.38
Probability of addiction to ENDS	X04_YV1124: Likeliness of someone becoming addicted to e-cigarettes or other electronic nicotine products (1 = Very unlikely, 2 = Somewhat unlikely, 3 = Neither likely nor unlikely, 4 = Somewhat likely, 5 = Very likely)	0.0037	2.04
Frequency of visiting convenience stores, small markets, or liquor stores	X04_YX0182: In past 30 days, how often visited a convenience store, small market, or liquor store (1 = Not at all, 2 = Once in the past 30 days, 3 = 2-3 times in the past 30 days, 4 = Once a week, 5 = 2-3 times per week, 6 = Almost every day)	0.0033	1.82
Self perception of overall health	X04_YX0088: Self perception of overall health (1 = Excellent, 2 = Very good, 3 = Good, 4 = Fair, 5 = Poor)	0.0028	1.53
Perception of nicotine as the primary cause of cancer from smoking cigarettes	X04_YC9120: Believe nicotine is the chemical that causes most cancer from smoking cigarettes (1 = Definitely yes, 2 = Probably yes, 3 = Probably not, 4 = Definitely not)	0.0026	1.39
Perceived duration of smoking shisha or hookah tobacco before health harm occurs	X04_YH1180: How long you think someone has to smoke shisha or hookah tobacco before it harms their health (1 = It will never harm their health, 2 = Less than a year, 3 = 1 year, 4 = 5 years, 5 = 10 years, 6 = 20 years or more)	0.0023	1.26
Perceived ease of purchasing tobacco products in a store for people your age	X04_YX0499: How easy you think it is for people your age to buy tobacco products in a store (1 = Very easy, 2 = Somewhat easy, 3 = Somewhat difficult, 4 = Very difficult)	0.0022	1.20
Widespread disapproval of ENDS	X04_YX0012: General perception: Most people disapprove of e-cigarettes or electronic nicotine products (1 = Definitely yes, 2 =	0.0020	1.11

© 2023 Le TTT. *JAMA Network Open.*

	Probably yes, 3 = Probably not, 4 = Definitely not)		
Perceived harmfulness of nicotine in nicotine replacement products	X04_YX0759: Harmfulness of nicotine in nicotine replacement products such as nicotine patch, nicotine gum, nicotine inhaler, nicotine nasal spray, lozenge, or pill (1 = Not at all harmful, 2 = Slightly harmful, 3 = Somewhat harmful, 4 = Very harmful, 5 = Extremely harmful)	0.0018	0.99
