# Supplementary material for: Key Risk Factors Associated With Electronic Nicotine Delivery Systems Use Among Adolescents
Source: JAMA Netw Open. 2023 Oct 20;6(10):e2337101. doi: 10.1001/jamanetworkopen.2023.37101 (PMC10589803; doi:10.1001/jamanetworkopen.2023.37101)
Supplement: Supplement 2. — Data Sharing Statement [file jamanetwopen-e2337101-s002.pdf]

Data Sharing Statement

Data
Data available: Yes
Data types: Deidentified participant data
How to access data: https://www.icpsr.umich.edu/web/NAHDAP/studies/36231
When available: beginning date: 01-01-2013

Supporting Documents
Document types: None

Additional Information
Who can access the data: Available everyone
Types of analyses: Most types of statistical analyses
Mechanisms of data availability: signed data access agreement
